# Mesoporous Bioactive Glass Combined with Graphene Oxide Quantum Dot as a New Material for a New Treatment Option for Dentin Hypersensitivity

**DOI:** 10.3390/nano10040621

**Published:** 2020-03-27

**Authors:** Sung-Ae Son, Dong-Hyun Kim, Kyung-Hyeon Yoo, Seog-Young Yoon, Yong-Il Kim

**Affiliations:** 1 Department of Conservative Dentistry, Dental Research Institute, Pusan National University, Yangsan 50612, Korea; 2 R&D Center, DAEWON MATERIALS Co., Ltd., 365, Sinseon-ro, Nam-gu, Busan, 48561, Korea; kimnano75@gmail.com; 3 School of Materials Science and Engineering, Pusan National University, Busan 46241, Korea; seweet07@pusan.ac.kr (K.-H.Y.); syy3@pusan.ac.kr (S.-Y.Y.); 4 Department of Orthodontics, Dental Research Institute, Pusan National University, Yangsan 50612, Korea; 5 Dental and Life Science Institute, Pusan National University, Yangsan 50612, Korea

**Keywords:** graphene oxide quantum dot, mesoporous bioactive glass, dentinal tubule, hypersensitivity

## Abstract

Dentin hypersensitivity is one of the most common clinical conditions usually associated with exposed dentinal tubules. The purpose of this study was to identify the potential of a graphene oxide quantum dot coating for mesoporous bioactive glass nanoparticles as a new material for the treatment of dentin hypersensitivity by investigating its mineralization activity and dentinal tubules sealing. Mesoporous bioactive glass nanoparticle was fabricated by modified sol-gel synthesis. X-ray diffraction was performed to characterize the synthesized nanoparticle Fourier transform infra-red spectroscopy investigated the functionalized surfaces. The distribution of the specific surface area and the pore size was measure by Pore size analysis. The morphology of sample was observed by Field Emission Scanning Electron Microscope (FESEM) and Field Emission Transmission Electron Microscope (FETEM). After disk-shaped specimens of mesoporous bioactive glass nanoparticles and graphene oxide quantum dot coated mesoporous bioactive glass nanoparticles (n = 3) were soaked in the simulated body fluid for 0, 1, 5, 10,and 30 days, the amount of ions released was observed to confirm the ionic elution for mineralization. Sensitive tooth model discs (n = 20) were applied with two samples and evaluated the dentinal tubule sealing ability. The spherical mesoporous bioactive glass nanoparticles and graphene oxide quantum dot coated mesoporous bioactive glass nanoparticles with a diameter of about 500 nm were identified through FESEM and FETEM. The ion release capacity of both samples appeared to be very similar. The amount of ion released and in vitro mineralization tests confirmed that graphene oxide quantum dot coating of mesoporous bioactive glass nanoparticles did not inhibit the release of calcium, silicon and phosphate ions, but rather that graphene oxide quantum dot promoted hydroxyapatite formation. In the FESEM image of the sensitive tooth disc surface, it was observed that graphene oxide quantum dot coated mesoporous bioactive glass nanoparticles sealed tightly the dentinal tubules. The graphene oxide quantum dot coating of mesoporous bioactive glass nanoparticles not only showed the excellent dentinal sealing ability but also rapidly promoted mineralization while minimizing the size increase by coating the mesoporous bioactive glass nanoparticles.

## 1. Introduction

Dentine hypersensitivity (DH) mostly occurs in patients between 20 and 50 years old. It causes an acute and short-duration pain [1]. According to the widely accepted hydrodynamic theory, the fluid within the dentinal tubules, which do not possess a smear layer, is subject to thermal, chemical, tactile or evaporative stimuli [2]. The movement of the fluid within the dentinal tubules stimulates the mechanical receptors which are sensitive to fluid pressure, resulting in the transmission of the stimuli to the pulpal nerves, ultimately causing pain response [2]. Pain can be reduced either by reducing fluid flow in the dentinal tubules or by blocking nerve reaction in the pulp. Attempts have been made to reduce fluid flow in the dentinal tubules by altering the smear layer (varnish, adhesive application), closing the tubules (amorphous calcium phosphate, calcium carbonate, bio-active-glass (BAG)), or by using desensitizing agents (potassium ions, oxalate, sodium fluoride) [3,4,5].

BAG has been introduced as promising biomaterials in the medical and dental fields. To date, BAG has been applied to various types of biomaterials, such as a bone or dentin scaffold, dental composite, and endodontic sealers. BAG releases calcium, sodium, and phosphate ions to raise the pH and create the precipitation of calcium phosphate and mineralization into hydroxyapatite [6,7,8,9,10]. Because of their buffering and apatite-forming abilities, BAG can be considered as great alternative for dentin hypersensitivity treatment [2,11,12,13,14,15]. However, BAG, which covered dentinal tubule, appeared to be removed easily by chemical and physical stimuli [16]. The recently introduced mesoporous bioactive glass (MBN) is found to be more effective for dentinal tubule obliteration and durability. In addition, MBN with the smaller size and larger surface area was much more effective for remineralization and dentinal tubule sealing [2,17,18]. 

Graphene is a honeycomb crystal lattice of carbon atoms bonded by sp2 hybridization, resulting in a lightweight and thin two-dimensional single atomic sheet. Because of the large surface area of Graphene, it is easily functionalized due to its higher electrical conductivity, excellent mechanical and physical properties. These features have led to the versatility in its usage in a range of biomedical areas. Graphene oxide (GO), an oxidative derivative of graphene with oxygen-derived free radicals, not only has excellent mechanical properties and a functionalizable surface, but is also highly hydrophilic and is well dispersed in aqueous solution [19,20,21]. Its structural properties allow it to be combined with polar organic/inorganic materials easily. The negatively charged functional groups of GO are known to react with Ca^+^ ions in the simulated body fluid (SBF) solution to induce nucleation of hydroxyapatite well. However, GO with the sp2’s plate structure is not appropriate to seal dentinal tubes. If a few nano-size graphene oxide quantum dot (GOQD) are combined adequately with MBN and many hydrophilic functional groups, this GOQD coated MBN nanoparticle could be used for a new treatment option for DH.

Unfortunately, no studies have so far used graphene oxide quantum dot in the treatment of hypersensitivity. Therefore, this study examined coating a graphene oxide quantum dot of nano-size with many hydrophilic functional groups in a mesoporous bioactive glass nanoparticle with a large surface area, synthesizing MBN@GOQD and, showing its mineralization activity and dentinal tubules sealing. The purpose of this study was to identify its potential as a material for the treatment of dentine hypersensitivity.

## 2. Materials and Methods 

### 2.1. Synthesis of Spherical Nanoparticle of Mesoporous Bioactive Glass (MBN)

MBN were synthesized by modified sol-gel method [2]. Briefly, in 150 mL of distilled water, 10 mL 2-ethoxyethanol (Sigma-Aldrich, St. Louis, MO, USA), 2 mL aqueous ammonia (Samchun, Seoul, Korea), 1.4 g calcium nitrate tetrahydrate (Ca (NO_3_) _2_.4H_2_O) (Sigma-Aldrich, St. Louis, MO, USA), 20 mL ethanol (Samchun, Seoul, Korea), and then 1 g hexadecyltrimethylammonium bromide (CTAB, Sigma-Aldrich, St. Louis, MO, USA) were added. After 30 min stirring, 5 mL and 0.25 mL of tetradecyl acetate (TEOS, Sigma-Aldrich, St. Louis, MO, USA), Triethyl phosphate (TEP, Sigma-Aldrich, St. Louis, MO, USA) were added sequentially and stirring for 4 h vigorously. The mole (Mol%) ratio of CaO: SiO_2_: P_2_O_5_ was 36:60:4. The white precipitates were obtained, dried at 60 °C for 24 h, and calcinated at 600 °C for 6 h with a heating rate of 1 °C min^−1^ and then furnace cooled naturally.

### 2.2. Synthesis of Spherical Nanoparticle of Graphene Oxide Quantum Dot Coated Mesoporous Bioactive Glass (MBN@GOQD)

The MBN@GOQD composite powder was synthesized using the colloidal processing method. GOQD (XFNANO Inc., Nanjing, China) suspension in distilled water and MBN suspension in the mixture of ethanol and distilled water in a 1:1 ratio were used. GOQD suspension was added dropwise to MBN suspension using a separatory funnel, with the weight ratio of 20:1. This process was performed slowly under magnetic stirring, and the mixture was dried in a vacuum oven at 60 °C.

### 2.3. Characterization of Composite Materials

The characteristics of the synthesized MBN and MBN@GOQD were analyzed by the following methods: Wide-angle X-ray diffraction (XRD, XRD, Rigaku, Ultima 4, Tokyo, Japan) analysis was measured at 40 kV, 40 mA with a range of 2θ from 10° to 60° and scanning speed of 1°/min. In order to identify the minimal phase of the specimen, small-angle XRD were scanned from 1° to 6° at a scanning speed of 1°/min. The chemical structure of the GOQD was measured using a Raman spectrophotometer (NRS-3300, JASCO, Tokyo, Japan) with a 532.24 nm wavelength. By Fourier transform infrared spectroscopy (FT-IR, PerkinElmer, Spectrum GX, Akron, OH, USA) with ATR method operating in the range of 2,000–400 cm^−1^, at the resolution of 4 cm^−1^ and the number of scans was 32 for each spectrum, the specimen’s functional groups and mineral formation were verified. Specimen morphology was observed by a 200 kV field-emission transmission electron microscope (FETEM, TALOS F200X, FEI, Hillsboro, OR, USA) operating at the accelerating voltage of 200 kV and chamber pressure of 4.0 × 10^−7^ mbar. Surface area and pore size analysis were measured by N_2_ adsorption-desorption isotherms and Brunauer-Emmett-Teller (BET) using an adsorption analyzer (Quantachrome autosorb-iQ MP, Quantachrome Instruments, Boynton Beach, FL, USA) at 77.35 K.

### 2.4. In Vitro Mineralization Ability and Ion Releasing Test

In vitro bioactivity test (apatite forming ability test) was analyzed in accordance with ISO 23317-07 [22]. MBN and MBN@GOQD powder (n = 3) were made into a disc with a diameter of 10mm and a thickness of 1.5mm. It was placed in the simulated body fluid (SBF) solution and kept for 0, 1, 5, 10, 30 days. After the SBF solution was removed, the sample was washed with the distilled water three times for 5 min. After drying it, the surface of the specimen was examined using XRD and FT-IR to analyze the formation of the hydroxyl-carbonate apatite, and the surface of the specimen was observed by FESEM (S-4700, Hitachi, Tokyo, Japan) operating at the accelerating voltage of 2 kV and chamber pressure of 9.634 × 10^−5^ Pa. After 0, 1, 5, 10, 30 days, MBN and MBN@GOQD extracts were collected, and the concentrations of calcium, silicon and phosphate ions in the solution were measured by inductively coupled plasma-optical emission spectrometry (ICP-OES) (Optima 8300, Perkin Elmer, Akron, OH, USA). Quality control standard 21(Perkin Elmer, Akron, OH, USA) for Ca and MISA-05-1(AccuStandard, Akron, OH, USA) for Si and P were used as calibration standard. Calibration ranges were 0.0983-10.0158 for Ca and 0.0996-10.0432 for Si, P.

### 2.5. Dentinal Tubule Sealing

The dentinal tubule sealing of MBN@GOQD were tested on sensitive dentin discs. Sensitive dentin discs with the thickness of 1mm were prepared perpendicular to the longitudinal axis of the teeth under the Enamel–Dentin Junction with a low-speed diamond saw (Struers Accutom-50, Ballerup, Denmark). The disc was polished using 320- and 600-grit silicon carbide polishing paper for 60 s each. Subsequently, the disc was soaked in 1 wt% citric acid solution for 20 s and thoroughly washed with water spray to open the dentinal tubules. This study was reviewed and approved by the Institutional Review Board of Pusan National University Dental Hospital (PNUDH-2019-023), 3 human premolars were collected and used. Sensitive dentin discs were applied with MBN@GOQD. Application protocol was as followed; Slurry prepared at a ratio of 100 mg/200 μL deionized water was applied to the dentin surface at a low speed of 15 s using a rotary cup and applied again for a total of 30 s.

After storing the applied discs for 14 days at 37 °C and 100% humidified atmosphere, the specimen were rinsed and dehydrated. FESEM assessment was performed to observe the changes in the exposed dentinal tubule.

## 3. Results

### 3.1. Sample Characterization

#### 3.1.1. Mesoporous Bioactive Glass Nanoparticles (MBN) and Graphene Oxide Quantum Dots (GOQD)

**Figure 1:**
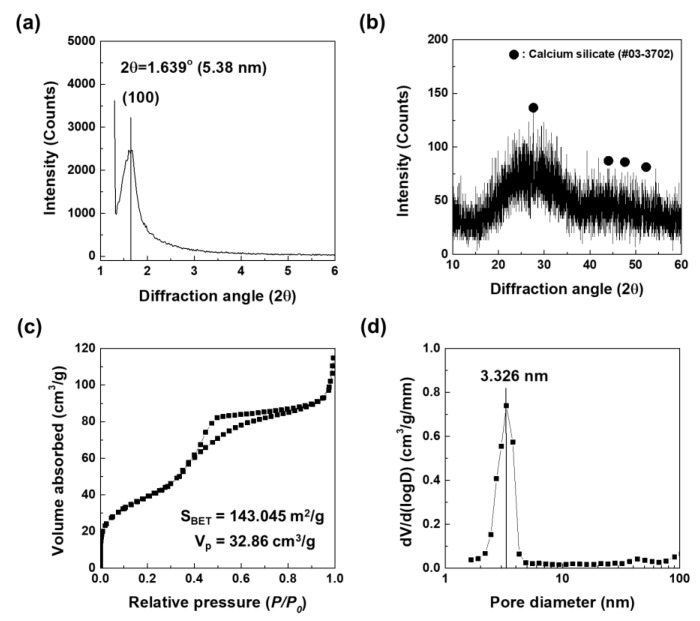
(**a**) Small angle, (**b**) wide angle X-ray diffraction (XRD) patterns, (**c**) N_2_ adsorption-desorption isotherms, and (**d**) pore size distribution of mesoporous bioactive glass nanoparticles (MBN).

Figure 1 shows the XRD patterns and N_2_ adsorption-desorption isotherms of as-prepared MBN. The small-angle XRD pattern of the calcined MBN samples are shown in Figure 1a. As shown in Figure 1a, one sharp diffraction peak (2θ = 1.639°, *d*-spacing = 5.38 nm) was indexed to the (100) diffraction peak. This peak indicated that MBN had well-ordered 2D-hexagonal mesoporous structures. As a result of wide-angle XRD in Figure 1b, MBN has also an amorphous state, indicating that calcium silicate phase has a glass phase with broad diffraction peak at 2θ = 15–35°. N_2_ adsorption-desorption isotherms were used to evaluate mesoporous structures of MBN. In Figure 1c, MBN represents type IV isotherm (exhibited characteristics of the type IV isotherm of mesoporous materials according to the IUPAC classification), which occurs when capillary condensation occurs due to the pore structure. The structure features a type H1 hysteresis loop. The feature is related to mesoporous materials with relatively uniform pores. As shown in Figure 1d, the pore size distribution of MBN showed a narrow distribution in which most pore sizes were close to the average of 3.326 nm.

**Figure 2:**
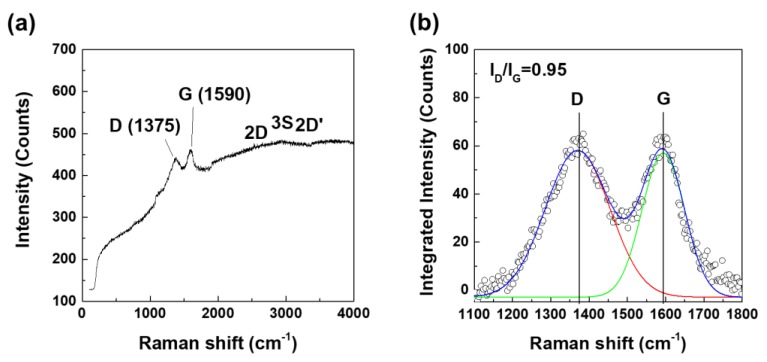
Raman spectra of GOQD; (**a**) wavelength range 400-4000 cm^−1^ and (**b**) pseudo-Gaussian peak-fitting results (1500–1900 cm^−1^).

Figure 2 shows the Raman spectra of the GOQD used in this study. In Figure 2a, the GOQD exhibited only two main peaks of 1375 and 1590 cm^−1^, which are associated with the D- and G-bands due to the oxygen-containing functional groups of graphene, respectively. As shown in Figure 2b, the integrated intensity ratio of the D/G bands (*I*_D_/*I*_G_ = 0.95) represents the degree of structural disorder of the GO.

#### 3.1.2. Graphene Oxide Quantum Dots Coated Mesoporous Bioactive Glass Nanoparticles (MBN@GOQD)

**Figure 3:**
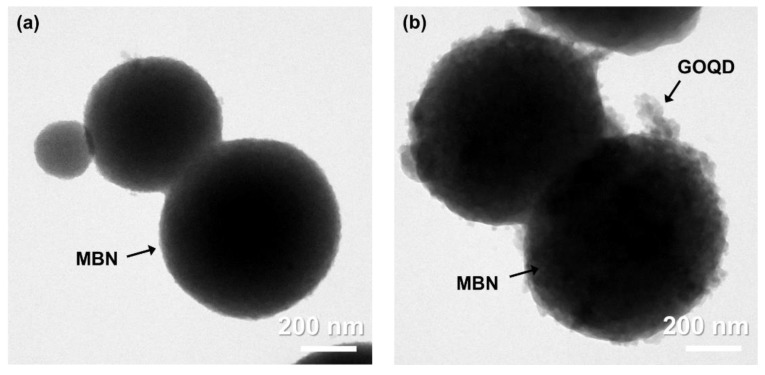
Field-emission transmission electron microscopy (FETEM) micrographs of (**a**) MBN and (**b**) MBN@GOQD.

In morphological and chemical structural comparisons of MBN and MBN@GOQD, the results of FETEM and FT-IR show obvious change. Figure 3 presents FETEM micrographs of the MBN and MBN@GOQD nanoparticles. Both MBN and MBN@GOQD had a diameter of about 500 nm. The spherical MBN with clean surfaces can be seen from images of the FETEM in Figure 3a. The graphene oxide quantum dot can be found coated in multiple layers on surface of MBN (i.e., MBN@GOQD), as shown in Figure 3b.

**Figure 4:**
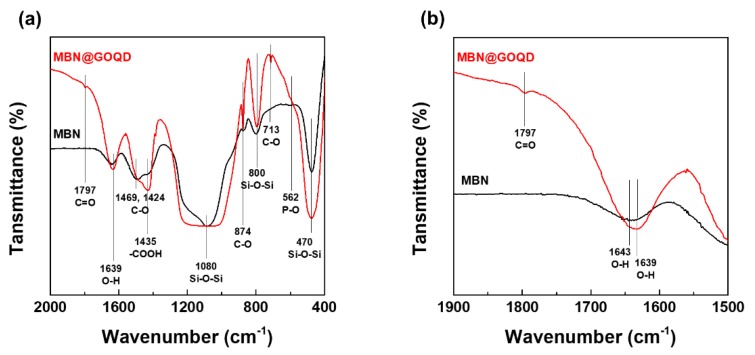
Fourier transform infrared spectroscopy (FT-IR) spectra of MBN and MBN@GOQD; (**a**) wavelength range 400–2000 cm^−1^ and (**b**) 1500–1900 cm^−1^.

Figure 4 shows the difference of chemical structure by FT-IR analysis in as-prepared MBN and MBN@GOQD samples. As shown in Figure 4a, the chemical structure of MBN and MBN@GOQD was compared using FT-IR spectroscopy. In the FT-IR spectra of MBN, Si–O–Si asymmetric stretching and rocking vibration is observed at 800 and 470 cm^−1^ [23]. The peak at 1080 cm^−1^ [23] is caused by Si–O–Si asymmetric stitching mode. The peak near 1643 cm^−1^ [6] is caused by the absorption of water from the surface Si-OH. The weak peak at 562 cm^−1^ appears to be the P–O band of phosphate group. The peak of 874, 1424, and 1469 cm^−1^ appears to be the C–O band of calcium carbonate [13].

However, the FT-IR result related GOQD in MBN@GOQD is different from MBN. In the case of GOQD of MBN@GOQD, the absorption bands at around 1797, and 1435 cm^−1^ corresponded to the stretching vibrations of C=O, –COOH, respectively. The absorption peaks at 1220–1250 cm^−1^ were attributed to C–OH, C–C stretching, and –OH bending vibrations [13]. As shown in Figure 4b, the stretching frequency (1643 cm^−1^) corresponding to O–H also shifted to 1639 cm^−1^ due to the chemical bonds of GOQD and MBN in MBN@GOQD sample [13,24].

### 3.2. In Vitro Ion Dissolution Test

**Figure 5:**
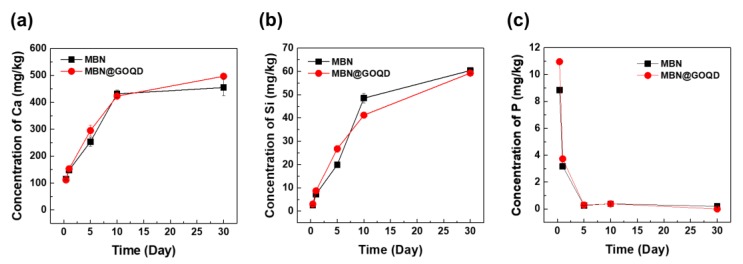
Ion’s concentration over time; (**a**) calcium, (**b**) silicon, and (**c**) phosphorus. Error bar means relative standard deviation.

The ionic elution of MBN and MBN@GOQD was shown in a very similar pattern as in Figure 5. The results of the concentration of calcium ion are shown in Figure 5a. The number of calcium ions increased continuously for 30 days in both MBN and MBN@GOQD, up to approximately 500 mg/kg. The number of silicon ions also continued to increase for 30 days in both cases, up to about 60 mg/kg in Figure 5b. However, the number of phosphate ions decreased continuously in contrast, resulting in very little elution after five days in Figure 5c.

### 3.3. In Vitro Mineralization Ability Test

#### 3.3.1. XRD Analysis

**Figure 6:**
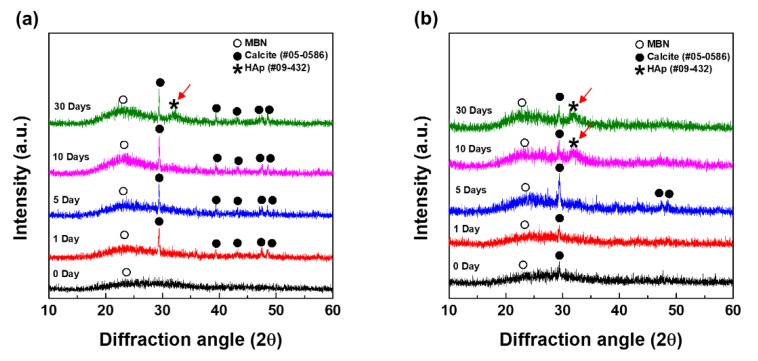
Wide angle X-ray diffraction (XRD) patterns of (**a**) MBN and (**b**) MBN@GOQD at 0, 1, 5, 10, and 30 days after soaking in the simulated body fluid (SBF) solution. Red arrows and asterisk (*) indicates hydroxyapatite peak (Ca_10_(PO_4_)_6_(OH)_2_, JCPDS # 09-0432).

Figure 6 shows the apatite-formation and calcite forming change that was obtained after the interaction of MBN and MBN@GOQD with SBF at various times. MBN and MBN@GOQD were reacted with SBF and then reacted for 0, 1, 5, 10, and 30 days and collected for XRD analysis. As shown in Figure 6a, calcite (calcium carbonate) peaks (CaCO_3_, JCPDS # 05-0586) were to be observed from MBN-reacted specimens. HAp (hydroxyapatite) peaks (Ca_10_(PO_4_)_6_(OH)_2_, JCPDS # 09-0432) were observed in the specimens reacted for 30 days. As a result of XRD of MBN@GOQD, calcite peak was present immediately after the reaction of SBF, and the peak of conversion to HAp began to be observed after 10 days. As shown in Figure 6b, calcite was formed immediately after the reaction in MBN@GOQD, and the formation of HAp was faster in MBN@GOQD than in MBN. Regarding the main XRD peak of calcite, the peak intensity of both MBN- and MBN@GOQD-reacted specimens generally showed a tendency to increase and then decrease for 30 days.

#### 3.3.2. FT-IR Analysis

**Figure 7:**
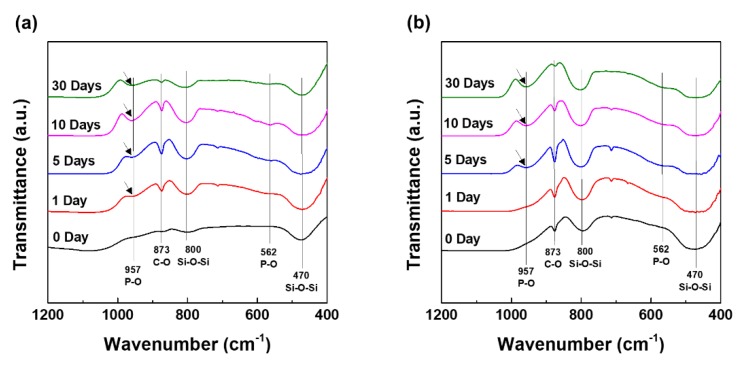
Fourier transform infrared spectroscopy (FT-IR) spectra of (**a**) MBN and (**b**) MBN@GOQD at 0, 1, 5, 10, and 30 days after soaking in simulated body fluid (SBF) solution.

Figure 7 shows the chemical structures of apatite-formation and calcite forming change that was obtained after the interaction of MBN and MBN@GOQD with SBF at various times. The change of chemical structures of MBN and MBN@GOQD were verified using FT-IR spectroscopy. In the FT-IR spectra of MBN, Si–O–Si asymmetrical stitching and rocking vibration is observed at 800 cm^−1^ and 470 cm^−1^ [13]. The peak [13] near 957 cm^−1^ may be caused by P-O bonding due to apatite-formation, while the peaks [13] near 873 cm^−1^ represent C-O peaks related to calcite. As shown in Figure 7a,b, the P-O bonding vibration was appeared early in MBN than in MBN@GOQD. In the absorption peak of calcite, the peak intensity of both MBN- and MBN@GOQD-reacted specimens was shown a tendency to increase and then decrease for 30 days. This result was also similar to XRD analysis.

#### 3.3.3. In Vitro Bioactivity and HAp Formation Process of MBN and MBN@GOQD

**Figure 8:**
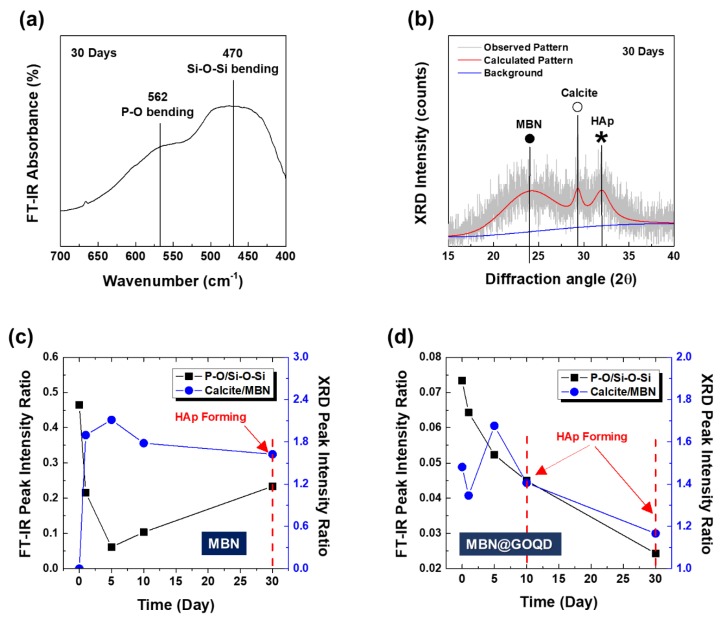
(**a**) FT-IR absorbance spectra, (**b**) calculated XRD pattern of MBN@GOQD with simulated body fluid (SBF) after 30 days, the in vitro bioactivity and HAp formation process of (**c**) MBN and (**d**) MBN@GOQD.

Figure 8 shows that the different in vitro formation process of HAp is compared for in vitro bioactivity and calcite formation changes of MBN and MBN@GOQD. The comparison is made by obtaining a ratio of peak intensities of FT-IR spectra and XRD pattern. As shown in Figure 8a, the in vitro bioactivity was determined with the peak intensity ratio of *I*_P-O_/*I*_Si_–_O_–_Si_. The formation change of calcite was determined with the peak intensity ratio of *I*_Calcite_/*I*_MBN_ from the main peak of XRD [13]. Figure 8b presents the calculated XRD pattern of MBN@GOQD with SBF after 30 days.

As shown in Figure 8c, MBN sample showed a very high formation of calcite as in vitro bioactivity (i.e., *I*_P-O_/*I*_Si_–_O_–_Si_) decreased at 5 days, and HAp was formed at 30 days as formation of calcite (i.e., *I*_Calcite_/*I*_MBN_) was gradually degraded with increasing in vitro bioactivity [25,26]. The data of Figure 8c suggest the correlation between HAp formation and the degradation of calcite in the in vitro interaction of bioactive glass with SBF. Moreover, it was found that the HAp formation of bioactive glass increased with the effect of the degradation behavior of calcite in SBF. In contrast, MBN@GOQD had the highest calcite formation at 5 days, similar to MBN, but HAp was formed at 10 days earlier than MBN with gradual decrease of in vitro bioactivity. MBN@GOQD also showed a constant gradual decrease of in vitro bioactivity (Figure 8d).

**Figure 9:**
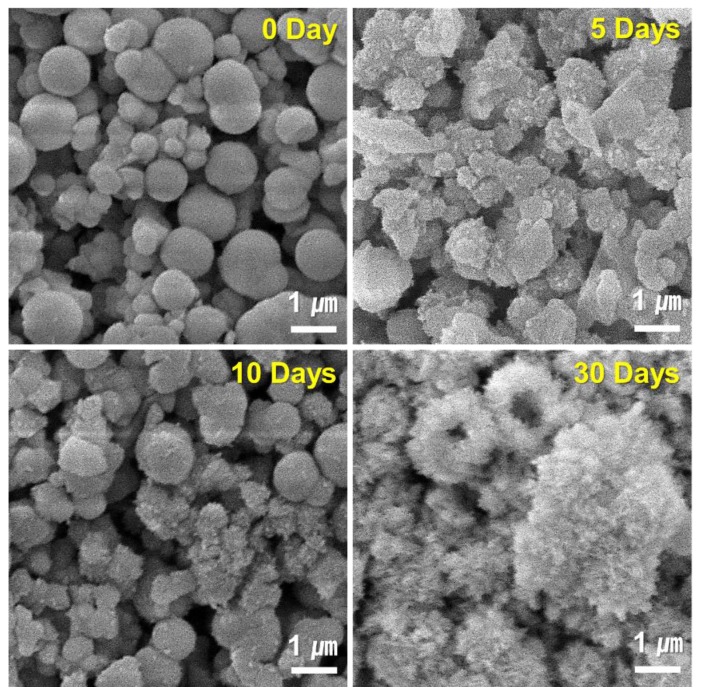
Field-emission scanning electron microscopy (FESEM) images of MBN at 0, 1, 5, 10, and 30 days after soaking in simulated body fluid (SBF) solution.

**Figure 10:**
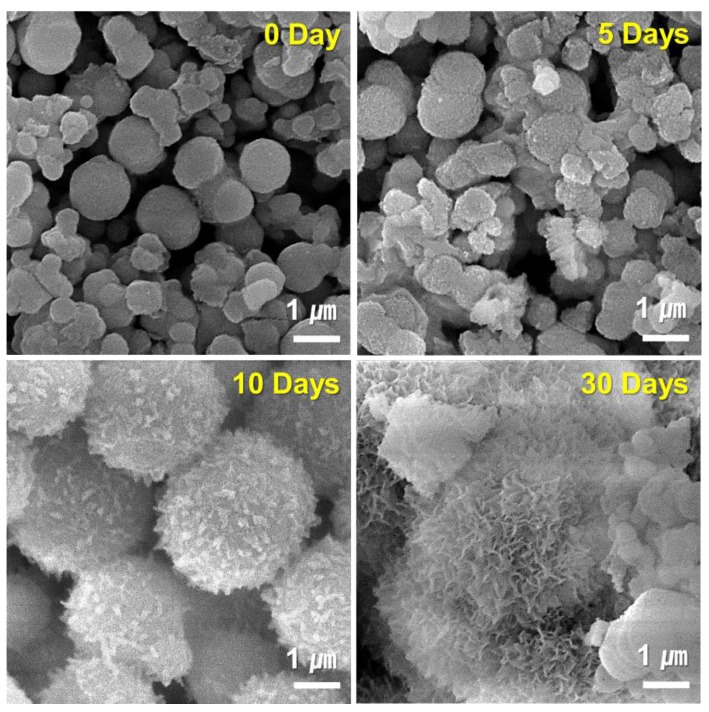
Field-emission scanning electron microscopy (FESEM) images of MBN@GOQD at 0, 1, 5, 10, and 30 days after soaking in simulated body fluid (SBF) solution.

Figure 9 and Figure 10 show the results of FESEM images after responding to MBN and MBN@GOQD to the SBF solution at various times. In the FESEM image, calcite appeared in the form of clusters on the surface of the bioactive glass particles, and hydroxyapatite was observed in the form of flakes or needle shape. As shown in Figure 9, SEM images of MBN specimens showed that calcite appears to be clustered in spherical particles after the reaction for 5 days. In the SEM image after 30 days, needle-shaped HAp was observed. As shown in Figure 10, results of the SEM image for MBN@GOQD specimen showed that calcite was formed in cluster form on the surface of spherical glass particles immediately after the reaction. Furthermore, it was observed that HAp was clearly produced in the form of needle-shape in 10 and 30 days after the reaction. These morphology results were confirmed with results from Figure 6, Figure 7 and Figure 8. 

### 3.4. Dentinal Tubule Sealing

**Figure 11:**
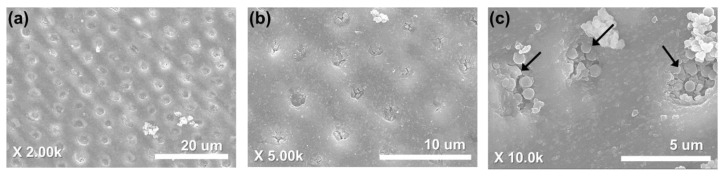
Field-Emission scanning electron microscopy images of MBN@GOQD after dentinal tubule sealing test for 14 days; (**a**) 2,000, (**b**) 5,000, and (**c**) 10,000 magnification. Red arrows indicate MBN@GOQD particles in the dentinal tubule.

In the FESEM image of the sensitive tooth disc surface, which was applied by MBN@GOQD, it was observed that MBN@GOQD sealed uniformly the dentinal tubules (Figure 11a,b). The magnified image after 14 days at 37 °C, 100% humidified condition shows that MBN@GOQD tightly sealed the dentinal tubule (Figure 11c).

## 4. Discussion

BAG as one of DH treatment modelities has been studied for a long time. Ion dissolution, osteoconductivity and apatite forming ability of BAG promotes remineralization on the dentin surface through mechanically occluding dentinal tubules [2,11,13,14,17]. In some previous observations, incomplete closure of the dentinal tubule was observed. In addition, BAG powder has delaying effect to partially hinder the calcium and phosphate ionic deposition [18]. In this study, one of attempts to improve BAG’s inherent drawback was to use GOQD. We synthesized MBN@GOQD, which adds the merits of GOQD to MBN, to apply to the new treatment option for dentin hypersensitivity. The spherical MBN and MBN@GOQD with a diameter of about 500 nm were identified through FESEM and FETEM. It was also possible to identify the surface of MBN with GOQD coating. XRD and FTIR confirmed that the formation of calcium carbonate during the synthesis of MBN@GOQD. Calcium carbonate was formed by calcium ions released from the crystal as an intermediate reactant during the formation of hydroxyapatite. And the surface of GO is rich in hydrophilic functional groups such as -O and -OH. They react strongly with calcium ions to provide a favorable environment for the formation and growth of hydroxyapatite crystals [27,28]. Furthermore, the adsorption and desorption isotherm graph show that MBN has a uniform pore with isotherm of type IV isotherm. After synthesizing MBN, the MBN surface area was 143.045 m^2^/g and the total pore volume was 32.86 cm^3^/g. The pore size distribution of MBN showed a narrow distribution in which most pore sizes were close to the average of 4.73 nm. Given the results of FESEM and FETEM, MBN@GOQD was synthesized in a spherical form, which is suitable for closing dentinal tubules. The GOQD coating used in this study didn’t increase the size of MBN. In this study, we controlled the size of spherical MBN for the proper sealing based on our previous study [2]. Jung et al.’s study showed that MBN with the larger surface area using a hexadecyl trimethyl ammonium bromide (CTAB) template was much more effective for remineralization and dentinal tubule sealing [2,17,18]. Synthesized MBN and MBN@GOQD have a suitable form to be applied for dentinal tubule obliteration and remineralization. Furthermore, the application of MBN@GOQD powder with 500 nm size on tooth disc model demonstrated the complete dentinal tubule sealing. The MBN@GOQD used in this study can be considered as suitable for the sealing of the dentinal tubule well while minimizing the size increase by coating the porous MBN.

MBN and MBN@GOQD can be applied not only into the dentinal tubules of the crown area, but also dentinal tubules of the root area. Since the root of the tooth is covered by gingival tissue and alveolar bone, MBN and MBN@GOQD can be considered such as bone graft materials, which are required to be tested on the stricter standard. In this study, bioactivity tests for MBN and MBN@GOQD were performed in accordance with ISO 23317:2014. ISO 23317:2014 specifies a method for detecting apatite formed on a surface of a material in simulated body fluid (SBF). ISO 23317 includes biomaterials such as dental implant, bone graft materials and others. Newly developed biomaterials intended for bone replacement must be tested in vitro with SBF [22]. If the MBN and MBN@GOQD can induce and promote the formation of a hydroxyapatite on the surface layer when immersed in SBF solution, each analysis (XRD, FTIR, ICP-OES and FESEM) can investigate the bioactivity and apatite forming ability.

From ICP-OES analysis, even though GOQD is coated to mesoporous, the ion release capacity of MBN and MBN@GOQD appeared to be very similar. Calcium ion levels continuously increased up to 30 days in both MBN and MBN@GOQD, up to about 500 mg/kg, and silicon ions continued to increase up to 30 days in both MBN and MBN@GOQD, up to about 60 mg/kg. In the process of synthesizing MBN@GOQD, the surface of MBN was filled and coated with GOQD to convert the pore size and surface shape into a non-porous form. This result suggests that MBN@GOQD, which has sufficient ion release, is likely to remineralize dentinal tubules, as Groh et al. showed in their study [29].

In vitro test was performed to verify the mineralization in MBN@GOQD in accordance with ISO 23317:2014. From XRD and FT-IR analysis of MBN@GOQD, calcium carbonate peaks were present immediately after reaction with SBF solution, and peaks interpreted as HAp (Ca_10_(PO_4_)_6_(OH)_2_, JCPDS # 09-0432) began to be observed 10 days after the reaction. 

To compare in vitro bioactivities and calcite formation changes between MBN and MBN@GOQD, we adopted ratios of peak intensities of FT-IR spectra (*I*_P-O_/*I*_Si_–_O_–_Si_), where I_P-O_ refers to the intensity of the P–O bending vibration around 562 cm^−1^ and *I*_Si_–_O_–_Si_ refers to the intensity of the Si–O–Si bending vibration at 470 cm^−1^ [23] and the peak intensity ratio of *I*_Calcite_/*I*_MBN_ from the main peak of XRD. From the analysis, MBN sample showed a very high formation of calcite as in vitro bioactivity (i.e., *I*_P-O_/*I*_Si_–_O_–_Si_) decreased at 5 days, and HAp was formed at 30 days as formation of calcite (i.e., *I*_Calcite_/*I*_MBN_) was gradually degraded with increasing in vitro bioactivity. This analysis showed their correlation between HAp formation and the degradation of calcite in the in vitro interaction of bioactive glass with SBF. Moreover, it was found that the HAp formation of bioactive glass increased with the effect of the degradation behavior of calcite in SBF. These results were also confirmed by our previous study [13]. In contrast, MBN@GOQD had the highest calcite formation at 5 days, similar to MBN, but HAp was formed at 10 days earlier than MBN with gradual decrease of in vitro bioactivity. MBN@GOQD also showed a constant gradual decrease of in vitro bioactivity. This is based on the fact that GOQD is partially coated on the MBN surface, so that the bio-chemical reaction between MBN surface and SBF is also constant. Therefore, MBN@GOQD shows that formation of HAp was increased by the coating of GOQD. The functional role of GOQD with negatively charged surface is to provide favorable sites for HAp formation [27,28]. This result is also consistent with a study by Liu et al. that GO promotes HAp formation [27]. The results of ion release and in vitro mineralization test showed that MBN@GOQD has superior remineralization ability than simple bioactive glass.

After 5 days, cluster-type calcite was observed in spherical particles in the FESEM image of MBN. Needle-shaped HAp was observed after 30 days. However, in the case of MBN@GOQD, clustered calcite was observed immediately after the reaction, and needle-shaped HAp was observed clearly after 10 days, which is earlier than MBN. This result is consistent with the results of XRD and FT-IR, suggesting the possibility of the earlier obliteration of dentinal tubules by the rapid formation of HAp when MBN@GOQD is applied to dentinal tubules under body fluids. Figure 11 confirmed that MBN@GOQD was remineralized after 14 days at 37 °C under 100% humidified condition and sealed uniformly the dentinal tubules.

Unfortunately, this study compared mineral formation activity only in vitro and did not take into account the pulpal pressure and acidic challenge that should be considered in practical applications. For one of dentin hypersensitivity treatment modalities, further study should include permeability test to determine whether the material effectively seals dentinal tubule. Nevertheless, this study synthesized graphene oxide quantum dots with size-controlled mesoporous bioactive glass for the first time to produce biomaterials and controlled them. Furthermore, it is the first attempt to confirm that increased mineralization activity in SBF solution. This study confirmed the remineralization and sealing ability of MBN@GOQD; future studies will address the possibility of manufacturing it in the form of various types of dental products and its potential in clinical application.

## 5. Conclusions

When MBN@GOQD was synthesized, spherical nanoparticles were formed and GOQD was uniformly coated in the mesoporous of MBN. The dentinal tubule sealing was acquired from its application on a sensitive tooth disc. Ion releasing and in vitro mineralization ability tests confirmed that GOQD coated MBN did not inhibit the release of calcium, silicon and phosphate ions, but rather that GOQD promoted HAp formation. As a result, MBN@GOQD not only has the excellent physical sealing ability but also promotes mineralization. It could be considered that the combination of graphene oxide and bioactive glass could be one of treatment modalities for new hypersensitivity treatment option.
